# Viral viability markers of SARS-CoV-2: a comparison of cell culture, genomic RNA RT-PCR, and subgenomic RNA RT-PCR

**DOI:** 10.1128/spectrum.00066-25

**Published:** 2025-09-08

**Authors:** Genoveva Cuesta-Chasco, Francesco Tommaso Aiello, Cristina Rodriguez, Anna Villasante, Juan Carlos Hurtado, Francesc Fernández Avilés, Luis Gerardo Rodríguez-Lobato, Mireia Navarro, Ignacio Grafia, Marta Bodro, Carolina García-Vidal, Alex Soriano, Maria Angeles Marcos

**Affiliations:** 1 Department of Clinical Microbiology, Hospital Clínic of Barcelona-ISGlobal, University of Barcelona 16724, Barcelona, Spain; 2 Department of Infectious Diseases, Hospital Clínic of Barcelona-IDIBAPS, University of Barcelona 16724, Barcelona, Spain; 3 Department of Hematology, Hospital Clínic of Barcelona-IDIBAPS, University of Barcelona 16724, Barcelona, Spain; 4 Department of Oncology, Hospital Clínic of Barcelona-IDIBAPS, University of Barcelona 16724, Barcelona, Spain; 5 CIBER Epidemiologia y Salud Publica (CIBERESP), Instituto de Salud Carlos III 38176, Madrid, Spain; National Institute of Immunology, New Delhi, India

**Keywords:** subgenomic RNA, SARS-CoV-2, surrogate markers of active viral replication

## Abstract

Accurate methods to assess viral viability are crucial for determining isolation duration and antiviral therapy in immunocompromised patients. Although cell culture (CC) is the gold standard, it has limitations. Cycle threshold (Ct) values from genomic RNA (gRNA) RT-PCR and subgenomic RNA (sgRNA) RT-PCR have been proposed as markers of active viral replication. This study evaluated the correlation between CC, gRNA Ct values, and sgRNA to identify the best viral viability marker. This study aimed to evaluate the correlation between CC, gRNA Ct values, and sgRNA to identify the best viral viability marker. We conducted a prospective study on immunocompromised patients with suspected SARS-CoV-2 infection at a tertiary hospital (May 2021 to May 2023). Nasopharyngeal swabs were inoculated into Vero E6 cells and tested for gRNA using RT-PCR (Cobas 6800, Roche) and for sgRNA (non-commercial RT-PCR). The sensitivity (S), specificity (SP), positive (PPV) and negative predictive value (NPV), and accuracy were calculated using CC as the gold standard. Among 285 samples from 108 patients, gRNA RT-PCR had high S and NPV (1.0) but low SP (0.24) and moderate PPV (0.63). Ct analysis improved performance in extreme but not intermediate values. A Ct ≤ 30 maximized S but had low SP; Ct ≤ 25 yielded S (0.88), SP (0.89), PPV (0.92), NPV (0.84), and accuracy (0.88); sgRNA showed the highest S (0.99), SP (0.96), PPV (0.97), NPV (0.99), and accuracy (0.98). sgRNA detection is the best marker for identifying viable SARS-CoV-2, aiding decisions on isolation, antiviral treatment, or delaying chemotherapy in immunocompromised patients.

**IMPORTANCE:**

Identifying whether a patient still has contagious SARS-CoV-2 is essential for managing isolation, antiviral treatment, and other clinical decisions—especially in immunocompromised individuals. While viral culture is the gold standard for confirming viral viability, it is slow, expensive, and not widely available. Many hospitals rely on RT-PCR tests, but these detect viral genetic material whether or not the virus is still active. This study shows that detecting subgenomic RNA (sgRNA), a molecule only present when the virus is actively replicating, is a highly accurate way, a molecule only present when the virus is actively replicating, is a highly accurate way to determine whether the virus is still viable. Compared to standard PCR or viral culture, sgRNA testing better predicts who is truly infectious. These findings support sgRNA as a useful tool to guide clinical management and infection control in vulnerable patients.

## INTRODUCTION

The SARS-CoV-2 pandemic has underscored the critical need for rapid, accurate, and accessible methods to assess viral viability and infectiousness across a wide range of clinical contexts. While real-time reverse transcription polymerase chain reaction (RT-PCR) remains the primary tool for detecting viral genomic RNA (gRNA), the mere presence of viral genetic material does not reliably distinguish between active infection and non-viable viral remnants (1).

This limitation has important implications for both clinical management and public health, particularly in decisions related to isolation, hospital discharge, or the re-initiation of immunosuppressive therapies. In immunocompromised patients (such as those with hematologic malignancies, transplant recipients, or individuals receiving anti-CD20 therapies), prolonged viral RNA shedding with high viral loads is frequently observed. This condition may be associated with persistent symptoms. Immunosuppressive treatment is often halted due to these patients’ underlying disease. Moreover, these patients are primary candidates for antiviral therapy against SARS-CoV-2, as their weakened immune system leads to prolonged infection and increases the risk of viral mutations. Persistent infection not only delays treatment due to extended isolation requirements but also facilitates the emergence of novel viral variants (2).

Cell culture (CC) remains the gold standard for assessing viral viability. It is, however, hindered by long turnaround times, the requirement for biosafety level 3 laboratories (BSL-3), and the need for highly trained personnel (3). Moreover, any procedure involving viable viruses must be conducted under strictly controlled and safe conditions, thus limiting its applicability in routine clinical settings.

As an alternative, subgenomic RNAs (sgRNAs) have been proposed as surrogate markers of active viral replication. These viral messenger RNAs are transcribed only during ongoing viral replication and share common structural features: a 5′ leader sequence and a 3′ polyadenylated tail. SARS-CoV-2 produces multiple sgRNA species, and the choice of which to analyze greatly influences detection sensitivity and biological interpretation. For example, sgRNA derived from the nucleocapsid (N) gene is the most abundant but can persist even in the absence of an infectious virus, likely due to protection within intracellular vesicles (4–6).

In contrast, sgRNA from the spike (S) gene is highly prone to mutations, and other sgRNAs are often too short to be reliably detected. Among the different sgRNA species, the sgRNA corresponding to the envelope (E) gene has emerged as a particularly promising marker. It is more conserved across variants, more transient than N sgRNA, and more consistently associated with recent transcriptional activity.

The E protein, encoded exclusively from this sgRNA, is a key structural component that is highly conserved across all known SARS-CoV-2 variants and also present in SARS-CoV-1. It plays a critical role in the viral life cycle by inducing host cell lysis, modulating the host immune response, and facilitating virion assembly, budding, and release. Therefore, E sgRNA offers an optimal balance between detectability, stability, and specificity for active replication. This makes it a promising surrogate marker for assessing viral viability.

Several independent studies have demonstrated that E sgRNA correlates more strongly with clinical symptoms and active viral replication, thus supporting its potential as a reliable surrogate marker of viral viability (7, 8).

In this study, we aimed to validate the detection of E sgRNA as a practical and robust method for assessing SARS-CoV-2 viability in clinical samples.

## MATERIALS AND METHODS

### Patients and sample collection

A retrospective analysis of prospectively collected samples from immunocompromised patients with clinically suspected SARS-CoV-2 infection was performed between May 2021 and May 2023, at a tertiary referral university hospital in Barcelona. Immunocompromised patients were defined as oncology patients, solid organ transplant recipients, and those with hematological diseases.

Nasopharyngeal swab samples were collected following standardized clinical protocols established at our institution, in accordance with international guidelines (9). All samples were placed in a viral transport medium consisting of Hanks’ balanced salt solution supplemented with fetal bovine serum, antibiotics, and antifungals, as recommended for SARS-CoV-2 diagnostics. Samples were transported and stored at 4°C immediately upon arrival at the laboratory.

The time from sample collection to processing was kept below 24 h for all specimens to minimize RNA degradation and preserve viral viability, as supported by previous studies (1, 10).

### Microbiological methods

#### Detection of SARS-CoV-2 gRNA RT-PCR and sgRNA RT-PCR

For the detection of gRNA by PCR, we used the commercial primers included in the automated Cobas 6800 system (Roche), according to the manufacturer’s instructions. This system targets two regions of the viral genome: the ORF1b gene and the E gene. Ct values were obtained retrospectively.

**Fig 1:**
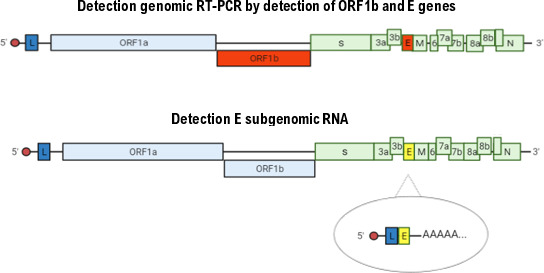
Diagram of the primers for the detection of genomic and subgenomic genes.

In contrast, sgRNA corresponds to viral messenger RNAs that share a common feature: the presence of a leader sequence at the 5′ end, identical to that found at the 5′ end of the viral genome, as well as a polyadenylated tail at the 3′ end. Based on this structure, the primers designed for sgRNA detection include, at their 5′ end, the leader sequence fused to the beginning of the target gene, and at their 3′ end, a sequence complementary to the poly(A) tail and the 3′ end of the gene (Fig. 1).

Total nucleic acid was extracted from samples using the MagNAPure Compact system (Roche, Switzerland). All the samples were analyzed for the presence of sgRNA of the E gene using the primers and PCR parameters described by Santos Bravo et al. (11).

Samples with a Ct value > 40 were considered negative.

#### SARS-CoV-2 CC

The samples were cultured within 24 h after collection. VERO E6 monkey kidney epithelial cells (ATCC CRL-1586) were used for SARS-CoV-2 culture. Samples were considered positive if a characteristic cytopathic effect (CPE) was observed, marked by the appearance of small syncytia that increased in size and number until the entire monolayer was affected. Positive results were then confirmed by indirect immunofluorescence using a specific monoclonal anti-SARS-CoV-2 antibody (CERTEST, Spain). Viral culture was considered negative in the absence of a CPE 10 days after inoculation.

#### Statistical analysis

A diagnostic validation study design was used to assess how the qualitative and quantitative Ct of RT-PCR gRNA and sgRNA RT-PCR correlate with CC as a gold standard for viability. The results were collated in a 2 × 2 contingency table identifying true positive (TP), false positive (FP), true negative (TN), and false negative (FN) results to calculate the sensitivity (S), specificity (SP), positive (PPV) and negative predictive value (NPV), and accuracy along with the 95% confidence intervals (CIs). To assess the precision of the agreement, 95% CIs were calculated for the kappa values, with 0.8 and above representing very good agreement. The statistical analysis was performed using R software (version 4.2.1) and the appropriate R packages for data visualization and statistical testing.

## RESULTS

During the study period, 285 NPS were collected from 108 immunocompromised patients with a median of 2.54 (1–3) samples per patient. CC, gRNA RT-PCR, and sgRNA RT-PCR were performed in all of the samples, but the Ct of gRNA RT-PCR was not available in 13 samples. In 61 cases, the CC was contaminated with fungi precluding its interpretation and being excluded from the analysis.

Using CC as the reference standard, the correlation with gRNA RT-PCR and sgRNA RT-PCR was studied. Additionally, considering that previous studies used the Ct value as a guide to monitor infectiousness, we analyzed the correlation of CC with gRNA RT-PCR results when the Ct value was ≤25 and ≤30, based on previously published literature (12, 13). Table 1 shows the TP, FP, TN, and FN values.

**TABLE 1:** Results of RT-PCR gRNA, Ct value of the RT-PCR gRNA, and RT-PCR sgRNA according to the cell culture result^*a*^

Cell culture	RT-PCR gRNA		Cutoff Ct 25 of RT-PCR gRNA^*b*^		Cutoff Ct 30 of RT-PCR gRNA^*b*^		RT-PCR sgRNA	
	P	N	≤25	>25	≤30	>30	P	N
Negative (*n* = 99)	75 (FP)	24 (TN)	9 (FP)	63 (TN)	34 (FP)	38 (TN)	4 (FP)	95 (TN)
Positive (*n* = 125)	125 (TP)	0 (FN)	103 (TP)	12 (FN)	115 (TP)	0 (FN)	124 (TP)	1 (FN)
Total (*n* = 285)	200	24	112	75	149	38	128	96

^*a*^ FN, false negative; FP, false positive; N, negative; P, positive; TN, true negative; TP, true positive.

^*b*^ In 13 cases, the Ct was not available.

From these results, the S, SP, PPV, and NPV of gRNA, sgRNA, and for Ct 25 and Ct 30 cutoff points of gRNA. All values were reported with their corresponding 95% CI. The results are presented in Table 2.

**TABLE 2:** Performance of RT-PCR gRNA, Ct cutoff values of 25 and 30, and RT-PCR sgRNA to detect positive culture^*a*^

Test	S	SP	PPV	NPV	Accuracy
RT-PCR gRNA	1 (1–1)	0.24 (0.16–0.33)	0.63 (0.56–0.69)	1 (1–1)	0.67 (0.6–0.73)
RT-PCR sgRNA	0.99 (0.98–1)	0.96 (0.92–1)	0.97 (0.94–1)	0.99 (0.97–1)	0.98 (0.96–1)
Cutoff Ct 25	0.89 (0.84–0.95)	0.88 (0.79–0.95)	0.92 (0.87–0.97)	0.84 (0.75–0.92)	0.88 (0.84–0.93)
Cutoff Ct 30	1 (1–1)	0.53 (0.41–0.64)	0.77 (0.70–0.84)	1 (1–1)	0.8 (0.76–0.87)

^*a*^ NPV, negative predictive value; PPV, positive predictive value; S, sensitivity; SP, specificity.

The Cohen’s kappa was *κ* = 0.524 for gRNA, *κ* = 0.946 for sgRNA and *κ* = 0.765 and *κ* = 0.577 for the Ct 25 and 30 cutoffs, respectively, indicating a higher level of agreement between the sgRNA and the CC compared to the other tests.

Table 2 shows that using both the gRNA and a Ct cutoff of 30 as a marker of viral viability resulted in a high S but low SP. In contrast, applying a Ct cutoff of 25 demonstrated a good S, SP, PPV, and NPV. However, the use of sgRNA provided the highest S, SP, PPV, and NPV.

The relationship between different strata of RT-PCR Ct values of gRNA and the CC was evaluated. The samples were divided into four groups according to the Ct, and the results are shown in Table 3. Both extreme values, ≤20 and >30, adequately correlated the positive and negative CC results, respectively. However, the intermediate values showed a worse predictive performance.

**TABLE 3:** Distribution of culture and sgRNA results by Ct RT-PCR of gRNA strata

*Ct*RT-PCR gRNA	*N*	Viral culture		
		Positive	Negative	% of positive/negative cultures in each stratum
≤20	75	75	0	100/0
21–25	37	28	9	75.6/24.4
26–≤30	37	12	25	32.4/67.6
>30	38	0	38	0/100

## DISCUSSION

This study compared the performance of different detection methods as surrogate markers for viral viability in nasopharyngeal samples from immunocompromised patients with SARS-CoV-2 infection, highlighting the potential of sgRNA detection to overcome the limitations of CC as the gold standard.

Our results demonstrated that sgRNA exhibited both high S (0.99) and SP (0.96), suggesting that it is a superior surrogate marker for viral viability compared to gRNA, which, although highly S, showed a low SP (0.24). Notably, the SP of gRNA detection improved when considering a Ct cutoff of ≤25, particularly relevant in immunocompromised patients.

During the SARS-CoV-2 pandemic, several studies explored the association between Ct values in gRNA RT-PCR assays and viral infectivity, proposing Cts of 25 or 30 as markers for monitoring infectivity (14–16).

Generally, Ct values ≤ 25 or ≤30 were considered markers of active infection, whereas values > 30 suggested the presence of non-viable viral fragments. Lower Ct values, which are indicative of higher viral loads, were associated with more severe clinical outcomes and a higher likelihood of a positive viral culture (17, 18). Generally, Ct values ≤ 25 or ≤30 were considered markers of active infection, whereas values > 30 suggested the presence of no viable viral genome fragments. However, sole reliance on Ct thresholds can lead to FP or FN interpretations, a concern particularly pronounced in immunocompromised patients. Several studies have demonstrated that Ct values may not be reliable for monitoring infection progression in immunocompromised patients (4, 19–22).

SARS-CoV-2’s genomic structure includes two large genes (ORF1a and ORF1b) that encode 16 non-structural proteins essential for viral replication and utilizes subgenomic messenger RNAs to express its major structural proteins (23, 24). Among these, the E protein, encoded by E sgRNA, is highly conserved protein across variants (even in comparison to SARS-CoV-1) and plays critical roles in host cell disruption, immune modulation, and virion assembly. Importantly, the presence of E sgRNA has been associated with active viral replication and the generation of infectious virions (7, 8).

Furthermore, E sgRNA is less prone to mutation across different variants and correlates more closely with clinical symptomatology than other targets (4, 18). Although some authors, such as of Alexandersen et al. (25), have questioned the correlation between sgRNA and viral viability, a growing body of evidence supports sgRNA as a robust marker of active infection (26, 27).

This study evaluated the performance of gRNA (qualitative), Ct cutoffs of ≤25 and ≤30, and sgRNA (qualitative) as surrogate markers of viral viability in SARS-CoV-2 infection, using CC as the gold standard. The gRNA RT-PCR demonstrated a high S (1.0) but low SP (0.24), resulting in a moderate PPV (0.63), a high NPV (1.0), and an overall intermediate accuracy (0.67). The Ct cutoff ≤25 provided a good profile with an S of 0.89, SP of 0.88, and accuracy of 0.88, while the Ct cutoff ≤30 maximized the S (1.0), but the SP was reduced (0.53). In contrast, sgRNA exhibited both a high S (0.99) and SP (0.96), with a high PPV (0.97) and NPV (0.99), resulting in a high accuracy (0.98). These results highlight the potential of sgRNA as a more reliable tool for assessing viral viability, demonstrating a stronger correlation with active viral infection compared to qualitative or quantitative (Ct values) RT-PCR gRNA, and addressing the limitations of CC as the gold standard.

In addition, in the analyses, the sgRNA assay is a qualitative RT-PCR test that demonstrates a high correlation with CC results, making it particularly useful for managing antiviral therapy and isolation in immunocompromised patients. The analysis of samples with Ct values ≤ 20 showed a strong correlation with viral viability, since all these samples were positive for both CC and sgRNA. In contrast, samples with Ct values > 30 were consistently negative for both CC and sgRNA, suggesting that the Ct is a reliable predictor of viral viability only at these extreme values. However, in the intermediate range of Ct values (21 and ≤30), there was greater variability in the results concerning CC. Although a Ct cutoff of 30 is recommended for immunocompromised individuals to prioritize a high NPV, this can lead to prolonged antiviral therapy, which may not be free from adverse events and carries significant economic costs. Given these considerations, it would be advantageous to routinely perform both gRNA and sgRNA RT-PCR assays in this patient population, particularly since the latter is an easily implementable technique.

We observed four cases with positive sgRNA RT-PCR but negative viral culture. We consider these to be likely false positives, although the overall sensitivity of the sgRNA assay remains high, as described above. It is also important to note that, while viral culture is considered the gold standard, it has its own limitations (3) (e.g., variability in sensitivity and dependence on sample handling). Due to limited sample volume, we were unable to repeat the culture in these cases to confirm whether technical failure may have occurred.

Conversely, we encountered one case with a positive viral culture and negative sgRNA result. We believe this was likely due to technical failure in the sgRNA PCR.

One of the strengths of this study includes its large sample size, which to our knowledge is the largest in the literature in which viral culture has been systematically performed and compared to an alternative diagnostic method such as RT-PCR of sgRNA and the different Ct cutoff values of RT-PCR for gRNA. All the samples were processed immediately upon arrival at the laboratory, minimizing potential degradation or contamination. Additionally, this study was only focused on immunocompromised patients, addressing the specific challenges related to viral viability and infectivity in this vulnerable population.

However, this study has several limitations that need to be considered. First, its cross-sectional design prevents longitudinal assessment of viral dynamics over time and limits causal inference regarding the relationships observed. Second, our analysis focused primarily on virological methods; therefore, we did not collect detailed clinical data such as treatment regimens, symptom progression, or patient outcomes, which could have enriched the interpretation of sgRNA detection. Finally, fungal contamination hindered the interpretation of the viral culture results among a subset of immunocompromised patients. The CC medium employed in our study contains antifungal agents designed to inhibit fungal overgrowth. Unexpectedly, upon analyzing the 61 samples where CC interpretation was hindered by fungal contamination, we found that all originated from only eight hematological patients. Consequently, it was not feasible to obtain interpretable CC results from any of these individuals. We hypothesize that these patients were receiving prophylactic antifungal therapy, potentially leading to the selection of fungal strains resistant to the antifungal agents present in the culture medium. This phenomenon has been documented in literature, where prolonged antifungal prophylaxis in hematological patients is associated with the emergence of resistant fungal pathogens. Due to the limited volume of clinical specimens and the stringent BSL-3 conditions required for handling SARS-CoV-2, repeating the CC experiments for these contaminated samples was not feasible. Importantly, the inability to assess viral viability through culture in this specific subgroup underscores the added value of subgenomic sgRNA detection, which served as the only feasible alternative for evaluating active viral replication in these cases. However, this limitation was partially mitigated by the inclusion of a large overall sample size, and the parallel use of sgRNA detection as an alternative marker of viral viability.

Although clinical data, such as symptomatology, disease progression, or treatment, were available for all patients, they were not included in this analysis because the objective of the study was strictly methodological: to evaluate the correlation between two molecular techniques (sgRNA and gRNA RT-PCR) and viral culture, considered the gold standard for assessing SARS-CoV-2 infectivity. This analytical focus allows the results to be interpreted independently of clinical variability and supports their potential applicability across diverse patient populations.

### Conclusion

In conclusion, the present results suggest that sgRNA detection is the best tool for identifying viable SARS-CoV-2 and assessing infectiousness. This is especially relevant in contexts in which this is critical for patient management, such as immunocompromised patients, in whom precise response is critical for decision making related to the duration of isolation, antiviral treatment, or delaying chemotherapy. Thus, the future development and promotion of commercial kits for sgRNA detection is necessary.
